# Stakeholder consultations on community-based rehabilitation guidelines in Ghana and Uganda

**DOI:** 10.4102/ajod.v1i1.1

**Published:** 2012-10-03

**Authors:** Mary Wickenden, Diane Mulligan, Gertrude O. Fefoame, Phoebe Katende

**Affiliations:** 1 Institute for Global Health, University College London, United Kingdom; 2 Sightsavers, United Kingdom; 3 Sightsavers, Accra Office, Ghana; 4 Africa Centre for Development Impact

## Abstract

**Background:**

The focus of this paper is the new broadened conceptualisation of community-based rehabilitation (CBR), which promotes the empowerment and inclusion of people with disabilities (PWDs) in diverse ways within their communities. New guidelines for CBR were launched in October 2010 by WHO/ILO/UNESCO/IDDC, and this paper describes part of the process by which these were produced using participatory approaches involving International Non-Government Organisations (INGOs) and local partners. The paper reviews the evolution of CBR and describes how grassroots consultation by INGOs working with key stakeholders in the disability arena can influence policy on disability issues, and reciprocally how policy change can inform organisations’ practice and research activities. This ongoing bidirectional influence is illustrated with data from the participatory consultation process about the new CBR guidelines carried out by Sightsavers in Uganda and Ghana

**Objectives:**

To consult with key stakeholders in the disability arena in Uganda and Ghana, in order to gain their opinions and suggestions for improvements to the then draft CBR guidelines, as part of a wider global participatory process of consultation on the document.

**Methods:**

The INGO Sightsavers gathered qualitative data through focus group discussions and questionnaires in both countries.

**Results:**

The participants’ critiques of the draft guidelines carried out in multiagency participatory processes were analysed thematically and fed back to the CBR guidelines editorial team.

**Conclusion:**

The paper concludes that stakeholders in diverse communities can actively contribute to shaping policy and practice through participatory consultations. Local and national government and non-government organisations and other key informants can inform the development of national and international guidelines and policies. This participatory approach can be successfully facilitated by INGOs. In turn, these processes have prompted organisations to adapt their own policies and programmes in order to be more responsive to the local needs and concerns of PWDs.

## Introduction

Community-based rehabilitation (CBR) for people with disabilities (PWDs) has been reconceptualised, and in recent years has moved to a cross-sectoral ‘social’ or ‘inclusive development’ model and away from a predominantly health focus. This paper describes part of the process of development of the new WHO/ILO/UNESCO/IDDC CBR guidelines launched in Abuja in 2010. Initially, it reviews the evolution of CBR from its earliest formulation to its present reconceptualisation as a framework and tool for inclusive development. It explains the principles underlying the new approach to CBR and how this links with the implementation of the UN Convention on the Rights of Persons with Disabilities (UNCRPD). It then describes two examples of the process of participatory consultations, led in this case by an INGO with local partners in Uganda and Ghana. It summarises the findings and discusses the ways in which INGOs can contribute both to the formulation of policy and the translation of this into practice, through participatory approaches.

### Problem statement

The gradual development of a new conceptualisation of CBR over the last decade was felt to require new guidelines to facilitate understanding and implementation of the strategy which aims to promote inclusive development. Disability-focused INGOs have been involved in the development and field-testing of the guidelines and in facilitating consultation processes worldwide. This is a new collaborative way of generating policy and practice documents and of ensuring that programmes and projects are well matched to the needs of stakeholders in the disability arena in their diverse community settings. This ambitious type of large-scale participatory process involving stakeholders globally has not been attempted before in the disability arena.

#### Aims of the study

The aim of this paper is initially to provide an historical overview of the origins and evolution of CBR in order to put the development of the new concept and guidelines in context. Then the paper describes the process and outcomes of the consultation led by the INGO Sightsavers in Uganda and Ghana, which contributed to the broader global participatory process of writing the CBR guidelines.

### Literature Review

This overview summarises the history of CBR from its inception to the present day. It describes the key ideas and initiatives which have influenced CBR’s development as a strategy. It discusses the current conceptualisation, its underlying principles, links with the UN Convention on the Rights of Persons with Disabilities (2007), the perceived need for new guidelines and their development using participatory methods.

#### Background

The last four decades have seen dramatic changes in conceptualisations of disability and in how citizens, civil society groups, service providers, government and NGOs perceive their contribution to ensuring the wellbeing of PWDs.^1^

Early approaches conceived disability as an almost exclusively health-related issue. Two early WHO Expert Committees on Medical Rehabilitation emphasised rehabilitation as an essential component of healthcare (WHO 1959, 1969). Countries both in the global ‘north’ and in the ‘south’ adopted conventional institutional systems of service delivery for PWDs through mainly urban rehabilitation centres and ‘care’ homes. In 1978, the Alma Ata Declaration, ‘Health for All’, obliged governments to consider rehabilitation in national plans for comprehensive healthcare. In light of these principles, in parallel with the concept of primary healthcare and in response to the lack of specialised medical rehabilitation services for PWDs in low income countries, the WHO began to articulate the concept of community-based rehabilitation (CBR) (Helander 1993, Boyce *et al*. 1997, Stone 1999, Finkenflugel, Wolffers & Huijsman 2005).

The aim of CBR is to ensure that rehabilitation services are provided to all PWDs, living in urban and rural settings and regardless of age and socio-economic status. This involves actions in the community using and building upon local resources as well as drawing on specialised secondary and tertiary services as appropriate. Gradually, the WHO recognised that most basic rehabilitation activities can be provided for people with disabilities in their own communities, using local resources and alongside primary healthcare, as well as other sectors. Fundamentally, the focus of CBR was on training people to carry out daily activities within their family and home contexts, participate in community activities, play and attend school or work. It emphasises using local resources and ‘low tech’ expertise available locally, rather than ‘high tech’ specialist services which are often not well adapted to the context, and are expensive or unavailable.

Echoing primary healthcare’s community health worker model, the cadre of staff who facilitate CBR by supporting and training PWDs and their families are CBR workers. After a short period of training (usually 3 to 6 months), they typically work within a limited geographical area or with a predetermined number of families, have access to information and support from a managing organisation or team, and may be either paid or voluntary. To facilitate their work and that of their managers, WHO published a CBR Manual, ‘Training in the community for PWDs’ (Helander *et al*. 1989). This took several years to develop, including the field-testing of draft versions. The manual consists of 34 modules: four guides and 30 training packages. These targetted local supervisors, community rehabilitation committees, PWDs and school teachers. The training packages were aimed at CBR workers and others who support people with a range of impairments (e.g. physical, intellectual, sensory, behavioural) and their families. The manual has played an important role in the promotion of CBR and in improving the quality of life of PWDs in the global south. It has been translated into over 50 languages, and is still used widely today.

However, during the 1980s, several key international initiatives and declarations served to reinforce the move of the CBR discourse away from purely medical model approaches focusing on the impairment and towards social and rights-based approaches. Key initiatives were: the World Programme of Action Concerning Disabled Persons (UN Enable 1982), The United Nations’ Standard Rules on the Equalisation of Opportunities for Persons with Disabilities (UN 1993) and the United Nations Decade of Disabled Persons (UN Enable 2006). Increasingly, disablement was seen not just as a health issue but as a social one.

These initiatives played a significant role globally, in promoting equalisation of opportunities and dignity for PWDs, and drove new domestic legislation in many countries in this direction. Furthermore, PWDs themselves increasingly demanded more active involvement in the planning of strategies and policies that affected their lives. Disabled People’s Organisations (DPOs) began playing significant roles in CBR initiatives and policymakers started to recognise the important roles PWDs themselves, their families and organisations could play in the quest for equality and human rights.

Despite these encouraging early signs of change, the prominence of ‘medical model’ discourses, which focus on what is different or ‘abnormal’ about the disabled person and seek to remediate this in the style of curative medicine, continued during the 1980s. However, in the USA, UK and elsewhere, radical shifts in thinking about the nature of disability were occurring (Swain *et al*. 1993, Oliver 1996, Barnes 2003). These then attributed the disabled person’s predicament not to their physical or psychological difference (impairment), but to society’s exclusionary and stigmatising treatment of them (Fine & Asch 1988, Shakespeare 1994). This much more politically aware and rights-driven ‘social model’ has spread globally and there has been a gradual shift towards using versions of this model to inform the provision of services across sectors including health and education, but also in law, social protection and employment. Subsequently, WHO developed a new framework to describe the important factors and relationships in disablement, the International Classification of Functioning, Disability and Health (ICF) (WHO 2001). This responds to the criticism that previous models had focused unduly on the nature of the disabled person’s individual (e.g. physiological, anatomical, psychological) differences (impairment) in comparison to a supposed ‘normal’ ideal. Thus disability has gradually separated itself distinctively and importantly from illness.

Although not universally accepted, and criticised for still being too ‘medical’, the ICF model was innovative in attempting a more clearly multidimensional view of PWDs’ situations. It thus set out to take account of political, socio-economic and environmental influences on their lives. There was recognition that medical approaches to rehabilitation which tend to focus on solely on cure and restoring ‘normality’ were unsatisfactory and that a comprehensive and holistic approach to PWDs’ needs was required. However, although shifting, the dominance of impairment-focussed ‘medical’ or individual models often still prevail today.

A global consultation in Finland in 2003 reviewed the progress of CBR in its 25th year with a broad caucus of stakeholders. Organised by WHO/ILO/UNESCO, it involved international organisations of and for PWDs and INGOs working in the CBR field. The most notable recommendations were to:

- promote CBR as a part of wider poverty-reduction strategies
- adopt a multi-sectoral approach and involve DPOs in CBR
- work to make disability part of international, regional and national agendas, e.g. through Poverty Reduction Strategy Papers (PRSP); Millennium Development Goals (MDGs) and the New Partnership for African Development (NEPAD).

To highlight these, the joint UN organisations updated the CBR Joint position paper (ILO/UNESCO/WHO 2004:2) and redefined CBR as: ‘A strategy for rehabilitation, equalisation of opportunities, poverty reduction and social inclusion of people with disabilities.’

The purpose was to promote human rights and a call for action against poverty, which was increasingly being recognised as often both causative of and resulting from disability (DFID 2000) The paper recognised that CBR is an effective strategy to meet the needs of PWDs. It noted that CBR needs to be implemented through the combined efforts of PWDs themselves, their families, organisations and communities, as well as the relevant governmental and non-governmental health, education, vocational, social and other services. Indeed in many low income countries, Ministries of Health and NGOs have come to play a vital role in promoting CBR. However, despite widespread anecdotal evidence that CBR is effective, the issue of large-scale, meaningful and comprehensive evaluation of it does remain problematic (Wirz & Thomas 2002, Cornielje, Velema & Finkenflugel 2008).

As CBR has evolved, it has not been without its critics, who suggest variously that it is: unworkable, overambitious, unrealistic or tokenistic in relation to, for example, empowerment issues (Miles 1989, 1996), or because of its reliance on volunteers or its financial unviability (Stone 1999), and that it is a second-rate solution or indistinguishable from community development (Lang 1999).

Although CBR is practiced in over 90 countries and is part of many national strategies, most programmes continue to follow a ‘vertical’ approach, focusing on one or two domains of life. For example, many focus on health alone, sometimes exclusively on physical rehabilitation. Others focus only on education or income generation. However, the single domain approach does not adequately address the multi-dimensional needs of PWDs and does not attempt to address the structural and societal exclusion they experience.

In order to ensure the relevance of CBR for PWDs, their families and the communities in which they live, it is now recognised that it must adopt a multi-sectoral comprehensive approach, addressing the key domains (or components) of well-being. The CBR Joint Position Paper promoted multi-sectoral and rights-based approaches, and importantly also focused on poverty reduction. However implementing such a multidimensional approach is complex (Barron & Amerena 2007). It was felt that putting the policy into practice required some guiding principles. The CBR Joint Position Paper thus identifed a need to develop ‘Guidelines’ for implementation of CBR (ILO/UNESCO/UNICEF/WHO 2004). The UN agencies agreed to develop these, with full collaborative stakeholder engagement. INGOs have played a major role in this process, as will be described below.

Also globally significant in the promotion of the rights of PWDs to equal recognition and participation, is the recent UN Convention on the Rights of Persons with Disabilities (UNCRPD) launched in 2006 (UN Enable 2006) and now signed by the majority of countries. This groundbreaking document produced with a participatory spirit, in collaboration with an international group of PWDs, promises to encourage and reinforce broader, more inclusive policies concerning and attitudes to all PWDs. Links between the UNCRPD and CBR are therefore expected to be close and crucial for the success of both. The new conceptualisation of CBR can be seen as a potentially powerful and effective tool for the implementation of the UNCRPD, especially in middle and low-income countries.

#### Current developments

The new CBR guidelines were drafted by the UN agencies, supported by 13 International NGOs and DPOs through the International Disability and Development Consortium (IDDC). Over 150 experts from diverse regions contributed and the draft was field tested in 25 countries (Khasnabis & Heinicke Motsch 2008).

**FIGURE 1:**
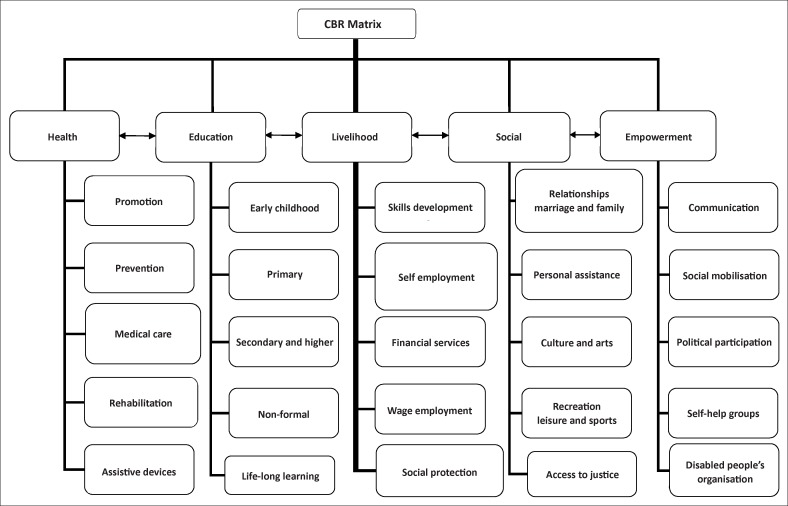
CBR Matrix (WHO 2006).

CBR is now conceived as having five major components: health, education, livelihood, social participation and empowerment, and these form the main chapters in the guidelines. In addition, there are sections on management of some special scenarios which are easily overlooked and which CBR needs to embrace including: HIV and AIDS, leprosy, mental health and crisis situations. Communities clearly vary – in terrain, demography, culture, political systems, socio-economic conditions and many other factors (Ingstad & Reynolds Whyte 1995). Therefore, there is no definitive model of CBR appropriate for all contexts. However, CBR programmes do have commonalities and there is a need for some basic principles to guide all and for a universal framework which will encourage and reflect a truly comprehensive multi-sectoral approach. To promote a holistic model of CBR, further work by groups of agencies working together was done to identify the key elements or sub-domains of the five key components, and this has resulted in the development of the ‘CBR matrix’ (Figure 1).

The matrix represents the domains which an effective CBR programme may need to consider, facilitate and or address directly, depending on local circumstances. It illustrates the sectors which combine to form a multi-sectoral CBR strategy. There are potential links both vertically and horizontally between these various suggested areas of action and focus. Although far from perfect, this model was arrived at through a consensus approach after consultation with a variety of actors.

Current thinking outlines that the goal of CBR is to facilitate and enable inclusive development and inclusive societies for PWDs. It focuses then on using mainstream local means and initiatives to promote wellbeing and life with dignity, such as healthcare from existing health facilities, education in regular schools and colleges, livelihood through traditional skills and local employment, income generation programmes, microcredit, inclusion and participation in local initiatives and community life. Thus the concept of ‘rehabilitation’ has been expanded from its previous clinical and impairment focus and is now seen to addresses all aspects of PWDs’s lives within their communities. CBR is designed to consider the needs of PWDs of all ages, so that families with a young disabled child are supported through early child development and inclusive education initiatives, whilst adults benefit from social, livelihood, citizenship and justice-focused activities. There is increasing recognition that poverty and disability are inextricably linked, although the exact nature of this relationship is still unclear (Coleridge 2007, Grech 2010, Barron & Ncube 2010). Therefore, many components of CBR aim to improve the economic wellbeing of PWDs. The formation of self-help groups (SHGs) and or Disabled People’s Organisations (DPOs) needs to be at the centre of the CBR strategy as described in the guidelines, to enable PWDs to gain equal access to mainstream opportunities (Hartley 2006).

The draft CBR Guidelines aim not to be prescriptive. They contain a range of experiences and real examples to promote and illustrate an up-to-date, practical strategy. Ways are suggested to achieve these aims through local initiatives that focus on inclusive development. The key approaches are to:

- meet basic needs and reduce poverty
- build capacity
- create opportunities for livelihood, health, rehabilitation, education and social life
- involve DPOs or facilitate PWDs to organise themselves
- collaborate across sectors in partnerships
- involve the whole community
- involve local government and leaders
- use the legislative, judicial and political systems.

The consultation process described below aimed to seek the views of stakeholders in the disability arena to ascertain their responses to the draft document and whether it was achieving its aims, and to gather suggestions for improvement before the final version was developed.

#### Background to the participatory consultation

The process of developing the new CBR guidelines was conceived as an exercise in multi-stakeholder cooperation Initially, a concerted effort was made to develop consensus on the CBR concept, its various components (domains) and elements (sectors). Various stakeholders including UN Agencies (ILO/UNESCO/WHO), representatives from member states, academics, NGOs, DPOs, professionals’ organisations and CBR experts met to finalise the outline of the guidelines and agree upon a common agenda called ‘Inclusive development to promote an inclusive society.’ It was agreed that CBR must use the principles of community action to ensure equality of access across sectors. Special efforts were made to ensure sufficient participation of PWDs, CBR practitioners and experts from the global south in the consultation process.

Thus the development and field-testing of the guidelines has been a participatory process. Approximately 30 CBR programmes in 25 countries contributed by reviewing the draft document. Feedback from this process was incorporated into the final edition and has provided critical analysis of the framework for CBR from a grassroots perspective and highlighted practical examples of good practice. The data reported here are from the consultation process in Ghana and Uganda that was facilitated by Sightsavers, an international NGO.

#### INGOs’ role in CBR and in the participatory development of the new guidelines

Many INGOs contributed to the process of developing the guidelines. We will describe the consultation facilitated by Sightsavers. Like many other disability-focused INGOs, Sightsavers, until recently, adopted vertical approaches, with a major focus on health interventions and an additional limited focus on primary education. Its work has traditionally been focused on eye healthcare, with smaller programmes for irreversibly visually impaired people to deliver daily living, orientation and mobility skills, or provide assistive devices. However, the Helsinki Review (WHO 2003) and the revised Joint Position Paper (ILO, UNESCO/UNICEF/WHO 1994) provided Sightsavers with a new framework, and in 2005 the organisation wrote a new internal CBR policy aligning its work within human rights, social inclusion and comprehensive approaches to inclusive development.

Alongside other INGOs, Sightsavers has been a major stakeholder in the development of the CBR guidelines, bringing considerable practical experience to inform their development. In Sightsavers’ particular case, their involvement has been as the lead author on the ‘social’ component, and as a member of the education component team.

In Uganda and Ghana, Sightsavers’ CBR programmes in collaboration with local organisations have used the original CBR manuals (Helander *et al*. 1989) extensively for many years. For example, Sightsavers assists with the funding of a degree course in CBR at the University of Education, Winneba, in Ghana, many of the modules of which were based on the 1981 manual. However, after a review in 2007, the course now reflects a more human rights-based approach to CBR, in addition to impairment-specific aspects. Here the need was very clear for an updated and reconceptualised set of guidelines about CBR.

In line with this new broader approach to disability and a participatory, human rights approach to CBR, Sightsavers were pleased to contribute to the production of the CBR guidelines by facilitating the consultations in Uganda and Ghana as described below.

#### Significance of the study

This paper is timely and important as it describes part of the process of development of the new WHO/ILO/UNESCO/IDDC CBR guidelines launched in Abuja in October 2010. It reviews the evolution of community-based rehabilitation (CBR) from its earliest formulation to its present reconceptualisation as a tool for inclusive development. It explains the principles underlying the new approach to CBR and how this links with the implementation of the UNCRPD. It then describes the process of participatory consultations led by an INGO with local partners in Uganda and Ghana. It summarises the findings and discusses the ways in which INGOs can contribute both to the formulation of policy and the translation of this into practice, through participatory approaches. The authors represent academia, an INGO and local partners in the two participating countries.

## Design and methods

### Materials

Materials used were draft versions of the guidelines and a semi-structured questionnaire and relevant documents for review.

### Sampling

Participants were recruited purposively through local networks in order to achieve as broad a spread of stakeholders as possible, representing a range of demographics, interests and experiences. Participants in both countries included: representatives from UN agencies, DPOs, INGOs and local NGOs, healthcare professionals, local government officials, itinerant teachers, academics and students from a CBR course, CBR personnel, PWDs, parents of disabled children, and religious and traditional leaders. In Ghana, 30 participants representing 17 organisations attended two national meetings in Accra, and approximately 300 people attended regional events. In Uganda, 55 stakeholders were invited and 48 participants from a range of backgrounds and organisations attended.

### Setting

Participatory consultation about the draft guidelines in Ghana and Uganda took place in the capital cities, Accra and Kampala respectively. In Ghana, two events took place in Accra, and subsequently nine events were held regionally. In Uganda there was an initial large plenary meeting and subsequently, over three days, smaller group meetings were held to critique the draft document in detail. A final plenary was held to summarise the groups’ findings.

### Design

This qualitative process of consultation was facilitated by Sightsavers staff in both countries and was essentially phenomenological in approach, drawing on the broad range of experiences of the participants and grounded in practical realities.

### Methods

Methods included focus group discussions about the draft guidelines and other locally relevant documents, the generation of case studies and completion of semi-structured questionnaires, which prompted feedback and suggestions for changes. Most of the discussions took place in English, although as the Sightsavers facilitators were local staff, they were able to translate and make notes in the local languages where necessary. Participants worked in groups to review and comment on each section of the draft guidelines.

### Analysis

Fieldnotes, transcriptions from the discussions and written material from the questionnaires formed the data presented here. This has been analysed thematically, initially by staff in the two fieldsites, and then a refining, clarifying and summarising process was undertaken by the authors (May 2011).

## Ethical considerations

The purpose, processes of the consultations and the methods to be used were explained verbally to the participants in advance. It was clear that participating in the consultation would have no implications for individuals or organisations, in relation to receiving services or involvement in future activities. The participants understood that information supplied would be used by third parties but that their individual contributions would be anonymous and that their comments and opinions would be presented as grouped data from their respective countries. The transcribed data were managed and stored securely by Sightsavers and by the WHO.

## Discussion of results

The findings of the analysis and related discussion are presented thematically here. Thirteen main themes emerged from the two field sites. Contributions from the two countries are presented together, although specified as to source where relevant. Unfortunately, verbatim quotes are not available for use in this paper and material from the different sources has been combined to present a coherent whole. Commentary and critical interpretation by the authors where relevant follows the country examples in each section.

Participants from both countries made rather similar general comments about the readability, clarity and usefulness of the document. They suggested that it was too long and detailed in places, that there was repetition, some difficult technical language and that a shorter ‘pocket’ version should be considered. They asked for ‘clearer definitions’, ‘more explanation’, ‘simpler language’ and ‘more examples’. The Ugandans wanted the guidelines to be more ‘prescriptive’ in places and with more practical ideas about implementation.

### Wider community engagement in the consultation process

Ghanaian participants thought that the consultation process should also have included opinion leaders and the wider community, in order to compare their responses with the outputs from the disability specific stakeholder focus groups so that wider, more practical approaches might be obtained. Linked to this, difficulties have been encountered in implementing CBR programmes because of lack of cooperation from the community members, persons with disabilities and their families. The challenges faced by local CBR personnel are often in engaging people to volunteer their expertise, skills and knowledge. Some of the solutions offered in this consultation included:

- building partnerships with people and organisations providing business development facilities because a CBR programme is interdisciplinary, multi-sectoral and cross cutting
- including everybody to achieve the set objectives.

The groups thought that the guidelines would give CBR workers insight about where, when and how to work with PWDs, especially about building partnerships with mainstream organisations.

More broadly, Ugandan participants suggested that CBR networking nationally and globally should be elaborated upon. Similarly to the Ghanaian comments above, they mentioned the need to create linkages between CBR, PWDs and all other social networks and community and development initiatives. The Ugandans also highlighted that people with some specific impairments were not adequately considered, for example, those with visual and hearing impairments, albinism and mental health difficulties and older people. They wanted more content about the needs of specific impairment groups.

This is an interesting finding, as there is a small body of literature, and more anecdotal evidence, that there are varying levels of exclusion and stigma across the different impairment groups, and also that there is often discrimination by some specific impairment groups towards others. Thus those who have physical or visual impairments are typically less excluded than those with cognitive, communication, behavioural or multiple impairments (Deal 2003). It is regarded as challenging in policymaking, inclusive development and in research to include these latter groups in ways which are not tokenistic and do not reinforce a potential hierarchy of exclusion across impairment groups.

### Livelihoods and lack of opportunities

In Ghana, the view was that PWDs are expected to accept any job without complaint, the perception being that they should be grateful for this. In low-income countries, scarcity of jobs is an issue for all unemployed people, disabled or not, and thus it is difficult for PWDs to compete in the job market. Participants felt that PWDs needed greater skills diversity, solidarity building, self confidence and the ability to speak as a united group on employment and skills issues.

Ugandans explained that the needs of PWDs as consumers of goods and services are often emphasised, whereas suppliers and providers also need to be made aware of disability issues in order to be able to provide equitable services.

### The role of culture and religion

In Ghana, the guidelines were judged to be culturally sensitive because the use of traditional medicine and herbal treatments were documented. Participants reported that they would largely use non-formal mechanisms for addressing grievances, because disability issues raised in official circles are subject to deeply ingrained societal stereotypes. It was felt that CBR workers needed to be able to analyse their local environments and cultural aspects carefully before suggesting interventions in such sensitive matters. For example, in most Christian communities, animal husbandry involving fowls and pigs would be successful, but in Muslim communities, suggesting the rearing of pigs would be inappropriate. It is important that communities themselves decide what is good for them.

After much deliberation, it was agreed that religion and culture played a major role in perpetuating expectations that PWDs should accept their impoverishment as divinely ordained and that many could not imagine a life different from their present one. This was attributed to strong cultural beliefs in most parts of the community that the birth of a disabled child is viewed as a curse. This in turn may hinder the development and inclusion of the disabled child in mainstream community life.

In Uganda, the potential cultural appropriateness of the guidelines was also highlighted. For example, the practice of children taking care of younger siblings was felt to be important. However, it was felt that catering for unique groups such as nomadic peoples and refugees and their specific ways of life need to be more thoroughly addressed in the guidelines. The issue of cultural rights to variation in practices also needed greater clarity in the guidelines to aid understanding.

### Gender

The Ugandan team observed that in the guidelines gender issues are focussed on women instead of on power dynamics between women and men and that gender should be addressed more clearly throughout the document..

In Ghana, they highlighted that the notion of marginalisation is not peculiar to disabled women alone, but rather a phenomenon associated with women generally. They suggested that this issue could be dealt with as a human rights violation.

### HIV and AIDS, sex and sexuality

In Ghana, the groups highlighted the issue of HIV and AIDS education for PWDs, and they raised the lack of cooperation from health personnel in receiving training about these issues in some cases.

The Ugandan team felt strongly that sex and sexuality need to be clearly addressed under the HIV and AIDS section of the guidelines. A stronger message about the needs of PWDs to access mainstream HIV and AIDS services was suggested, and the issue of HIV and AIDs sometimes being causative of impairment was raised.

### Involvement of PWDs and capacity building

Groups in both countries welcomed the importance given to communicating with and involving PWDs, particularly in relation to their communities and families. It was noted that this was missing from the original WHO CBR Manual (Helander *et al*. 1989). The Ugandan team argued that an emphasis on the disabled person being at the centre of processes could be put even more strongly, so that they were an active participant rather than passive recipient in CBR.

They felt that the roles of children and youths with disabilities were not given enough emphasis. Instead adults seem to be the category most targeted for increased participation in community activities and programmes. Interestingly, this criticism, of lack of sufficient attention to the needs of children, has also been made of the UN Convention on the Rights of PWDs (UN Enable 2007).

In addition, one of the key concepts considered missing in the ‘empowerment’ component was ‘counseling and guidance’. This was considered by participants to be essential in order to help PWDs to overcome the emotional and psychological effects of their impairments and to achieve confidence and self esteem and in turn to enhance group solidarity and dignity amongst persons with disabilities.

This has also recently been raised as an issue in the disability literature, though not particularly in developing country contexts. It is recognised that the psychological effect of discrimination and exclusion on PWDs can be serious and long-lasting, even in contexts where more visible physical or structural and political barriers have to a large extent been removed (Reeve 2006).

Both groups wanted to see greater emphasis on the importance of PWDs as role models. Participants agreed that when they have been active as role models in their respective communities and in CBR programmes this has really helped change the mindset of other PWDs in the community, as well perhaps as the attitudes of non-disabled people around them.

### Collective responsibility versus leadership

Within the self-help groups (SHGs) component, the development of leadership skills for all with disabilities was seen as a key element. However, the Ugandan team thought that a careful balance was needed between developing the skills of individual leaders and building the capacities of all members of SHGs and DPOs. It is important that these groups become empowered as a whole, rather than relying solely on key individuals.

Participants felt that self-help groups should develop on the basis of common needs and problems faced by disabled members in order to achieve collective responsibility and solutions. The participation of all in developing the group will ultimately lead to increased visibility of PWDs within the community and in turn to the development of individual members’ competencies and confidence.

### Lack of infrastructure

The Ugandans saw lack of infrastructure and accessibility as a challenge to CBR programmes, especially mentioning roads and transport. This presents a real barrier to reaching target groups for both service providers and users because PWDs are geographically dispersed and may be isolated. They felt that these issues were largely overlooked in the guidelines.

### Human resources

Some of the rehabilitation activities listed in the guidelines were perceived by the reviewers as specialised treatment requiring expert knowledge. As a result of the African ‘brain drain’ of healthcare professionals, some specialists are simply not available (Patel 2003). In Ghana for example, developing local expertise in working with people with severe developmental impairments has always proved difficult, even at the national level. Most general healthcare professionals do not receive training on disability issues, partly due to lack of funding, and partly to lack of political will. Healthcare course curricula were perceived as difficult to influence and change. Participants commented that the lack of adequate budget allocations at a local and national level was not addressed by the guidelines.

Similarly, the Ugandans said that the role of professionals in supporting CBR needed addressing and additionally that discussion about the role of volunteers and volunteering was also absent. In both countries, it was felt that both human and financial resources are lacking to support CBR.

### Beyond medical rehabilitation to rights-based approaches

Both groups welcomed the shift in the debate towards PWDs as rights holders and they underlined the benefits of human rights discourses and sustainable approaches. However, some participants reported that the concept of the rights-based approach was not clear. In Africa, in particular, it is also important to promote a move away from charity models, especially since many CBR programmes have been started by the churches. The Ghanaian team highlighted the omission in the guidelines of discussion about sensitising religious leaders about the importance of early childhood education for children with disabilities.

### Partnerships between groups and agencies

The Ghanaians recognised that at community level, partnerships between DPOs and CBR personnel were very important, especially in relation to provision of rehabilitation aids and appliances. DPOs are able to promote the sustainability of CBR through their ongoing involvement and participation. They also saw DPO partnerships with government as crucial, for instance in developing flexible educational curricula which are responsive to disabled children’s needs.

Beyond education, the Ugandan team stressed the role of governments as key implementers of CBR programmes and that partnerships between healthcare centres and DPOs were essential: for example, a participant who is the coordinator of an eye hospital unit commended the collaboration between the CBR programme and the unit. He reported that the CBR programme contributed a great deal to making outreach programmes successful and also getting clients prepared for both consultations and surgery. Additionally, CBR could be important in sensitising PWDs to register with the National Health Insurance Scheme to access better healthcare.

### Strengthening legislation

In Ghana, it was felt that various district assemblies could be active in strengthening existing domestic legislation to ensure inclusion of PWDs in all sectors of the economy. The Ugandan team would like further guidance on how to link CBR to the UNCRPD.

These contributions illustrate that many of the principles and key concepts in the CBR guidelines reflect the principles and provisions of the UNCRPD (UN Enable 2007). Undoubtedly, the guidelines will become an important tool for implementing the convention at the local level. As the groups in both countries hint, current legislation produces numerous institutional barriers. DPOs could serve as pressure groups to lobby governments who are not immediately willing to harmonise domestic legislation with the CRPD. The CBR guidelines have clear statements that can help promote respect for the persons with disabilities, and linkages between DPOs could build bridges between local, district, national, regional and international policy.

### Power and corruption

Both countries agreed that, as stated in the guidelines, political structures are very powerful. Some of the suggested activities in the draft document were considered controversial, given the political affiliations of some opinion leaders. They opined that since almost every decision made by political leaders affects local people, and that people with money often influence these leaders, subsequent decisions are not likely to favour the situation of persons with disabilities. Thus, underlying structural issues in many cultures may prove to be very challenging barriers to the development of real inclusivity.

## Limitations of the consultation process

As the participants were purposively sampled using the networks available to the field staff working with Sightsavers and other related programmes, it is possible that those participating did not represent the whole range of views which might have been held about the draft guidelines. Those who contributed were those who were able to come to the consultations. Others who were not able to attend because of lack of resources (time or money), access difficulties or for other reasons may have had different views. Additionally, group discussions are known to be more effective in collecting consensus data rather than individual opinions (Krueger and Casey, 2000) and it is possible that those with dissenting views may not have felt able to express these.

## Recommendations

There were a number of broad issues raised by either or both groups, which they suggest need further coverage throughout the guidelines or more specifically in other documents. These included: how to get started in CBR, detail on implementation, training and capacity building for PWDs themselves and or CBR workers, the need for further research, issues around scaling up and countrywide coverage, sustainability of programmes and CBR’s role in prevention of disability.

## Conclusion

A process of stakeholder consultation with a broad range of interested parties was facilitated in order to critique the draft version of the WHO/ILO/UNESCO/IDDC CBR guidelines through a participatory process facilitated by an INGO working with local partners in Uganda and Ghana. Qualitative data from this process were fed into the broader global consultation and thus contributed to the writing of the final version of the document.

The draft guidelines were also peer reviewed by cooperating universities and external experts, and the final CBR guidelines were published in October 2010, and their distribution globally in various languages and formats is currently in process. A concerted effort has been made to ensure broad ownership of the document and to develop it as a practitioners’ document for CBR managers. The momentum that has been created through this process will continue to grow. Further occasions for cooperation and knowledge sharing and additional opportunities for working in alliance across the spectrum of individuals and agencies are already being discussed and considered. One of these opportunities is to use CBR specifically and explicitly as a tool to implement the UNCRPD at grassroots level.

We conclude that key stakeholders including PWDs’ organisations and other key local stakeholders in developing countries can play a role in shaping public policy. These groups can use their own local experiences to inform and develop domestic and international policy in order to promote and secure the rights of PWDs. They can be assisted in these participatory processes by a number of other agencies, including international NGOs.

Sightsavers has launched a strategic plan 2009–2013 that has three out of four overall goals which can be located within the CBR guidelines and are aligned with the UN CRPD. We have demonstrated that the process of developing the CBR guidelines has used the experience of Sightsavers’ and other NGOs’ programmes to inform its content and ensure relevance in African contexts. Sightsavers’ grassroots research has been a means to influence policy on disability issues, and in turn, policy dating back to the Helsinki meeting has changed and informed Sightsavers’ research and practice.

The CBR guidelines are an important step forward in promoting CBR as an inclusive development strategy. Poverty is often the major barrier in improving inclusion and quality of life, accessing healthcare, education, housing, justice and other services. Accordingly, CBR will need increasingly to focus in cross-sectoral ways of improving access to basic human rights, working for the full inclusion, participation and wellbeing of PWDs. The new CBR guidelines focus on meeting basic needs for PWDs, accessing the benefits of mainstream developmental initiatives, and empowering PWDs and their families. They implicitly move disability away from its historical location solely within health to other sectors and encourage the implementation of the UNCRPD using community based initiatives. INGOs working in the disability sector have moved their approaches in response to and in parallel with these changes. CBR must increasingly operate as a rights-based and inclusive development strategy in order to ensure that the benefits of broader, mainstream development initiatives reach PWDs and their families. The new guidelines are an important tool to facilitate this strategy.

## Footnotes

1. We prefer the term ‘PWDs’, which reflects the social model of disability and the language adopted by the disability movement in the United Kingdom. This terminology is preferred because it is believed that societies disable people, not that disablement is an inherent part of the person. The term people with disabilities (PWDs) is used extensively in developing countries, and we do not intend to undermine this concept or people who favour this concept.
